# Clinical Super-Resolution Computed Tomography of Bone Microstructure: Application in Musculoskeletal and Dental Imaging

**DOI:** 10.1007/s10439-024-03450-y

**Published:** 2024-02-15

**Authors:** Santeri J. O. Rytky, Aleksei Tiulpin, Mikko A. J. Finnilä, Sakari S. Karhula, Annina Sipola, Väinö Kurttila, Maarit Valkealahti, Petri Lehenkari, Antti Joukainen, Heikki Kröger, Rami K. Korhonen, Simo Saarakkala, Jaakko Niinimäki

**Affiliations:** 1 Research Unit of Health Sciences and Technology, University of Oulu, POB 5000, 90014 Oulu, Finland; 2 Neurocenter Oulu, Oulu University Hospital, Oulu, Finland; 3 Medical Research Center, University of Oulu, Oulu, Finland; 4 Department of Radiotherapy, Oulu University Hospital, Oulu, Finland; 5 Department of Diagnostic Radiology, Oulu University Hospital, Oulu, Finland; 6 Department of Oral and Maxillofacial Surgery, Oulu University Hospital, Oulu, Finland; 7 Department of Surgery and Intensive Care, Oulu University Hospital, Oulu, Finland; 8 Cancer and Translational Medical Research Unit, Faculty of Medicine, University of Oulu, Oulu, Finland; 9 Department of Orthopaedics, Traumatology and Hand Surgery, Kuopio University Hospital, Kuopio, Finland; 10 Department of Applied Physics, University of Eastern Finland, Kuopio, Finland

**Keywords:** Super-resolution, Deep learning, Computed tomography, Cone-beam computed tomography, Musculoskeletal radiology, Dental radiology

## Abstract

**Purpose:**

Clinical cone-beam computed tomography (CBCT) devices are limited to imaging features of half a millimeter in size and cannot quantify the tissue microstructure. We demonstrate a robust deep-learning method for enhancing clinical CT images, only requiring a limited set of easy-to-acquire training data.

**Methods:**

Knee tissue from five cadavers and six total knee replacement patients, and 14 teeth from eight patients were scanned using laboratory CT as training data for the developed super-resolution (SR) technique. The method was benchmarked against ex vivo test set, 52 osteochondral samples are imaged with clinical and laboratory CT. A quality assurance phantom was imaged with clinical CT to quantify the technical image quality. To visually assess the clinical image quality, musculoskeletal and maxillofacial CBCT studies were enhanced with SR and contrasted to interpolated images. A dental radiologist and surgeon reviewed the maxillofacial images.

**Results:**

The SR models predicted the bone morphological parameters on the ex vivo test set more accurately than conventional image processing. The phantom analysis confirmed higher spatial resolution on the SR images than interpolation, but image grayscales were modified. Musculoskeletal and maxillofacial CBCT images showed more details on SR than interpolation; however, artifacts were observed near the crown of the teeth. The readers assessed mediocre overall scores for both SR and interpolation. The source code and pretrained networks are publicly available.

**Conclusion:**

Model training with laboratory modalities could push the resolution limit beyond state-of-the-art clinical musculoskeletal and dental CBCT. A larger maxillofacial training dataset is recommended for dental applications.

**Supplementary Information:**

The online version contains supplementary material available at 10.1007/s10439-024-03450-y.

## Introduction

Image quality plays a pivotal role in assessing musculoskeletal and dental pathologies. The most common modalities in the field include magnetic resonance imaging (MRI), radiography, ultrasound, and computed tomography (CT) [1–3]. While MRI provides excellent soft tissue contrast and radiography is widely available, CT imaging is the superior method for imaging changes in bone [2, 4, 5]. Clinical cone-beam computed tomography (CBCT) imaging devices can achieve a voxel size of up to 100–200 µm^3^ and are useful for detecting both orthopedic [6] and dental pathologies [7], joint trauma imaging [8], and radiotherapy planning [9, 10]. For example, CBCT has been recognized as the recommended modality for assessing wrist fractures [8, 11]. Despite the mentioned resolution, from the Nyquist’s theorem, the perceived *spatial resolution* is at least twice lower, and thus, the visible clinical features in CBCT can only be of 500 µm in size [12]. This, however, is not enough to observe bone microstructural changes. The CBCT image quality is limited by radiation dose, motion, acquisition geometry, receptor size, and the focal spot size of the beam. Quality assurance phantoms, that is, tissue-simulating test objects allow for assessing the technical image quality of a CT device. The modulation-transfer function (MTF) or task-transfer function can be calculated to quantify the spatial resolution of clinical CT [13, 14], and the resolution limit is approximately seven line pairs per centimeter [15]. In practice, a series of line pair patterns [13] or a high-contrast edge [16, 17] can be used to estimate the MTF. Other CT image quality parameters include the accuracy of CT numbers, uniformity, noise power spectrum, [15] and low contrast detectability [15].

The bone microstructure is conventionally seen only with laboratory micro-computed tomography (µCT) devices. For measurement in a clinical setting, CBCT is the most promising modality [18]. As an example, bone microstructural changes are known to be associated with osteoarthritis severity [19], and could be useful in the assessment of osteoporosis, bone strength and fracture risk [20, 21]. Detection of early osteoarthritis could facilitate earlier intervention, significantly reducing the socio-economic impact of the disease [22]. Karhula et al. have previously shown that bone subresolution features can be estimated with CBCT using texture analysis [23]. Individual quantitative parameters cannot be directly connected to local tissue changes but could be visible from high-quality images. Finally, dentomaxillofacial CBCT imaging requires high-image quality for multiple indications. The trabecular bone microstructure is one of the key factors for dental implant planning [24]. Dental and periodontal diagnostics [12], as well as assessment of ossicular chain and inner-ear pathologies [25], are all focused on assessing changes in tiny, mineralized structures.

One approach to increase image resolution is to improve upon the reconstruction technique. Recent advancements include iterative- [26, 27], model-based- [28], and learned [29, 30] reconstruction. However, these methods naturally require access to the raw CT projection images, access to which is typically restricted by the scanner’s manufacturer. Another method for upscaling could simply rely on image interpolation combined with antialiasing. However, such techniques have difficulties in removing artifacts and blur from the approximated high-resolution images [31].

Due to recent advancements in deep learning (DL), super-resolution (SR) methods can be used to predict impressive details from low-resolution images [32, 33]. They are based on convolutional neural networks (CNN) that either modify the original input image or generate entirely new images from latent space. High- and low-resolution images are used in the training process with different approaches. Unpaired training aims to match two datasets with different image quality without exact matches for each image [34, 35]. It is also possible to obtain only the high-resolution dataset and artificially distort the data to create matching low-resolution images [32]. Finally, the dataset could be collected using both low- and high-resolution imaging modalities and a subsequent co-registration. However, accurate co-registration is likely challenging in the case of highly distorted images.

Previously, SR has been used to increase MRI quality for the knee by Chaudhari et al. [36, 37]. The authors thoroughly evaluate the performance of the SR method for visualizing cartilage morphometry and osteophytes. Brain MRI SR has also been assessed for clinical image quality [38]. The first SR studies for inner-ear CBCT have been introduced using generative adversarial networks [39]. Finally, µCT imaging and SR have been used to assess bone microstructure in a preclinical setting [40]. Although CNN predictions could be explained by different interpretability methods [41–43], the DL applications are often criticized for their “black-box” nature. However, some deep learning SR algorithms are already on the market for CT [29, 44] and MRI [38]. Thus, guidelines and recommendations for thorough clinical validation of such algorithms are needed. Before clinical use of SR, it would be crucial to ensure that the CNN predictions only increase the image quality and do not add new or remove existing pathological features from the images [45].

In this study, we aim to enhance the image quality of clinical CBCT using a limited dataset of high-resolution laboratory µCT images. To assess the robustness of the method, the same framework is utilized for musculoskeletal and maxillofacial imaging, in areas with highly different tissue types. To provide a comprehensive understanding of the effects of the SR model, bone microstructure, technical image quality, and clinical image quality are assessed. We hypothesize that the SR methods trained with laboratory data can outperform conventional image processing for the quantification of bone microstructure, as well as technical and clinical image quality. Furthermore, while a robust SR algorithm might generalize to different musculoskeletal applications, we expect that additional dental data would be useful when training the SR algorithms in the maxillofacial application.

## Materials and Methods

### Training Data

**Fig. 1:**
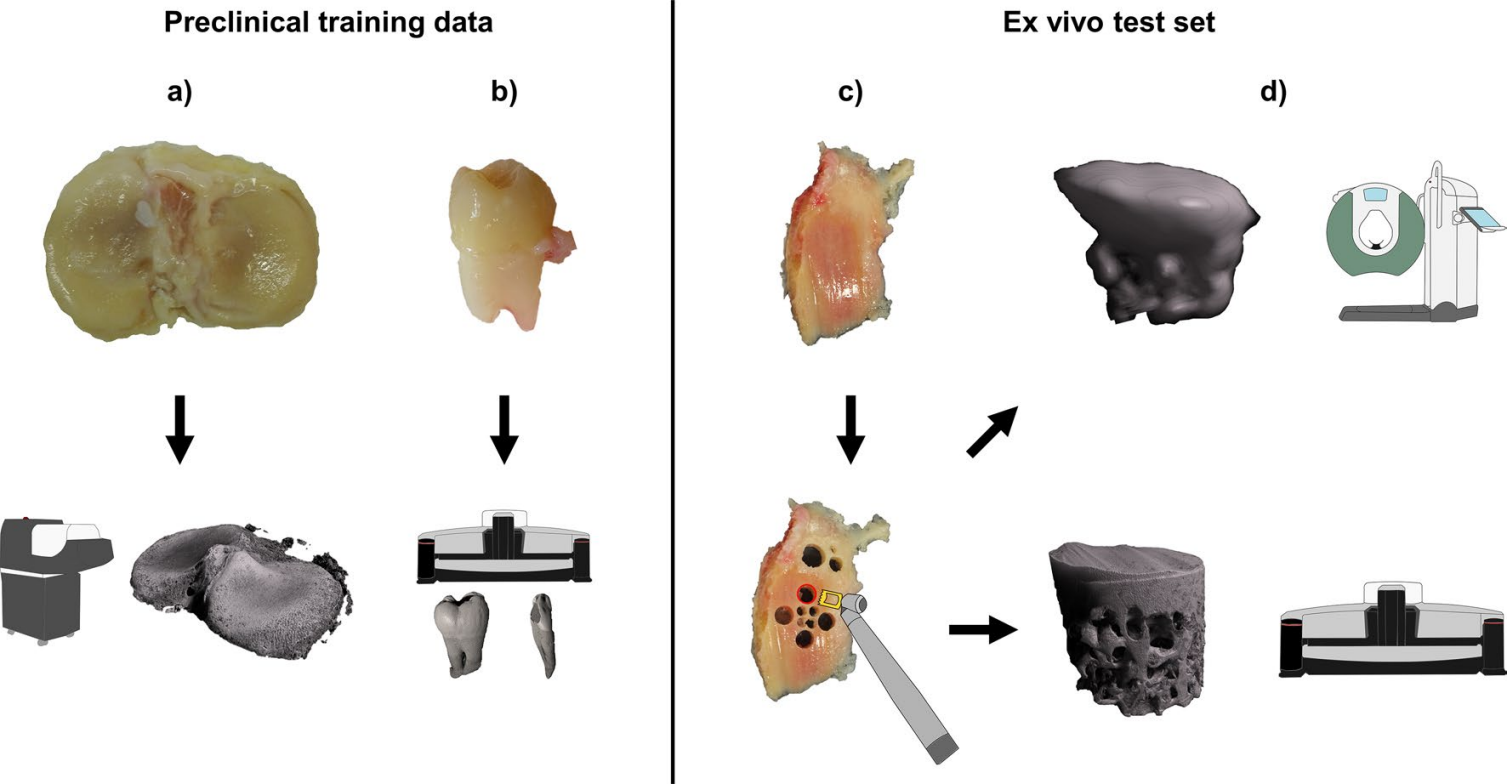
Training data (left) and ex vivo test set (right) acquisition. The full tissue blocks **a** were scanned using a preclinical micro-computed tomography (µCT) scanner (Skyscan 1176, resolution of 26–35 µm). Extracted teeth **b** were imaged using a desktop µCT (Skyscan 1272, resolution 19.8 µm). To obtain the ex vivo test set, small 4 mm osteochondral plugs were extracted (**c**). The plugs were imaged both with the desktop µCT (bottom right, Skyscan 1172, 2.75 µm resolution) and a clinical extremity cone-beam CT (CBCT) system (top right, Planmed Verity, 200 µm resolution) to provide realistic low- and high-resolution references (**d**). Note that due to the lower image quality, the cylindrical shape of the sample is distorted on the CBCT scan

The training data consist of twelve knee tissue block samples extracted from five healthy cadavers and six total knee arthroplasty (TKA) patients (Table 1). An overview of the image data acquisition is in Fig. 1. The sample harvesting was approved by the Ethical committee of Northern Ostrobothnia’s Hospital District (PPSHP 78/2013) and the Research Ethics Committee of the Northern Savo Hospital District (PSSHP 58/2013 & 134/2015). The tissue blocks are stored in phosphate-buffered saline after surgery, and subsequently imaged with a preclinical µCT scanner (Bruker Skyscan 1176; 80 kV, 125µA, 26.7 µm voxel size, 30–60 min scan time). The 1176 scanner has a scan bed with 68 mm diameter and 200 mm length, which is optimal for imaging the knee tissue blocks. The images were reconstructed using the manufacturer’s software (NRecon, beam hardening, and ring artifact corrections applied, 45–60 min reconstruction time).

**Table 1:** Dataset descriptions

Preclinical datasets	# images	# samples (n)	# patients (N)
Knee tissue blocks	220 544	12	11
Extracted teeth	45 540	14	8
Ex vivo test set	1 700	53	11
Clinical studies			
Ex vivo test set	1700	53	11
Wrist CBCT	313		1
Ankle CBCT	219		1
Knee CBCT	471		1
Dental CBCT	3 352		9
CT Quality assurance phantom	6		N/A

Samples from both total knee arthroplasty patients and asymptomatic cadavers were used in the preclinical training and test sets. Different patients were included for training and testing. The ex vivo test set was collected with both preclinical and clinical CT devices, and the characteristics are described in further detail by Karhula et al. [23]. Clinical studies were used to validate the method on realistic use cases

Furthermore, a total of fifteen human teeth were collected from nine patients with a tooth removal operation (Table 1, PPSHP 123/2021). The teeth were scanned using a high-resolution laboratory desktop µCT scanner (Skyscan 1272, Bruker Inc., Kontich, Belgium; parameters: 100 kV, 100 µA 19.8 µm voxel size, Cu 0.11 mm filter, 75–150 min scan time). The 1172 scanner allows scalable resolution with geometrical magnification, which is beneficial for imaging small individual teeth and osteochondral samples. The reconstruction was conducted using the Nrecon software (beam hardening and ring artifact corrections applied, 5 min reconstruction time). The reconstructions of fourteen extracted teeth from eight patients were used to provide further training data for the SR model in the case of dental CBCT. A tooth scan of one of the patients was excluded due to corrupted data in the µCT scan.

### Ex Vivo Test Set

To provide the ground-truth reference for bone microstructure prediction, we utilized a previously collected dataset [23] consisting of 53 osteochondral samples from nine TKA patients and two deceased cadavers without an OA diagnosis (Table 1; ethical approval PPSHP 78/2013, PSSHP 58/2013 & 134/2015). The samples were imaged using two devices: a clinical extremity CBCT (Planmed Verity, Planmed Inc., Helsinki, Finland; parameters: 80 kV, 12 mA, 200 µm voxel size, 20 ms exposure time) and a laboratory desktop µCT scanner (Skyscan 1272, Bruker Inc., Kontich, Belgium; parameters: 50 kV, 200 µA 2.75 µm voxel size, 2200 ms exposure time, 0.5 mm Al filter, 135 min scan time). The samples were imaged with the µCT one at a time, and with the CBCT scanner, a large batch of samples were imaged during one scan. The projection images were reconstructed with the corresponding manufacturer’s reconstruction software with a “standard” reconstruction filter applied for CBCT, and beam hardening and ring artifact corrections were applied for µCT (Nrecon, v.1.6.10.4, Bruker microCT, 20-70 min reconstruction time). The reconstructed volumes were coregistered to the same coordinate system using rigid transformations on the Bruker Dataviewer software (version 1.5.4, Bruker microCT).

### Clinical Images

The proposed method was further tested on clinical data acquired using the same Planmed Verity CBCT device (Table 1). The clinical dataset consists of one knee scan (50-year-old female; 96 kV, 8 mA, 200 µm voxel size, 10 s exposure time, “flat” reconstruction filter), one wrist scan (56-year-old female; 90 kV, 6 mA, 200 µm voxel size, 6 s exposure time, flat filter), and one ankle scan (34-year-old male; 96 kV, 8 mA, 400 µm voxel size, 6 s exposure time, flat filter). In the case of the knee and ankle, the imaging was done in the weight-bearing position. The participants are healthy volunteers, and the CBCT scans were acquired from the Oulu University Hospital digital research database. Finally, preoperative CBCT scans (Planmeca Promax; parameters: 120 kV, 5–6 mA, 200 µm voxel size, 8 s exposure time) were collected from the nine patients with tooth removal (ethical permission PPSHP 123/2021).

To validate the technical image quality, a commercially available CT quality assurance phantom (GE Healthcare, Model No. 5128754) was imaged using a diagnostic CT device (GE Revolution Frontier; parameters: 120 kV, 335 mA, 730 ms exposure time, 625 µm pixel size, 5 mm slice thickness, head filter).

### Super-Resolution Model

The training data were created from the preclinical tissue blocks by downscaling the µCT images. Three datasets with specific imaging resolutions used in the test images and the corresponding 4x magnifications were created, and a separate set of SR models were trained for each dataset (200 µm → 50 µm, 400 µm → 100 µm, 488 µm → 122 µm). First, µCT images were downscaled to the target resolution, and a Gaussian filter (kernel size = 7, *σ* = 0.5) was applied to mitigate aliasing artifacts. The input images were obtained by further downscaling the target images by a factor of four. To account for aliasing artifacts and simulate the lower imaging quality, this time Gaussian blurring (kernel size = 4, *σ* = 1) and median filtering (kernel size = 3) were applied after downscaling. The reconstructed image stacks were automatically divided into smaller 32 × 32 × 32 (input resolution) and 128 × 128 × 128 (target resolution) voxel patches suitable for training the SR models, resulting in thousands of training images (Table 1). The training data were augmented spatially using random rotations, translations, and flips. Furthermore, brightness and contrast were randomly adjusted, and random blurring was added to augment the grayscale values. Finally, the input and target volumes were randomly cropped and padded to match the network input and output size (16 × 16 → 64 × 64 for 2D, 16 × 16 × 16 → 64 × 64 × 64 for 3D models). The augmentations were based on our previously published SOLT library (https://github.com/Oulu-IMEDS/solt) and modified to account for the varying input and target image size.

**Fig. 2:**
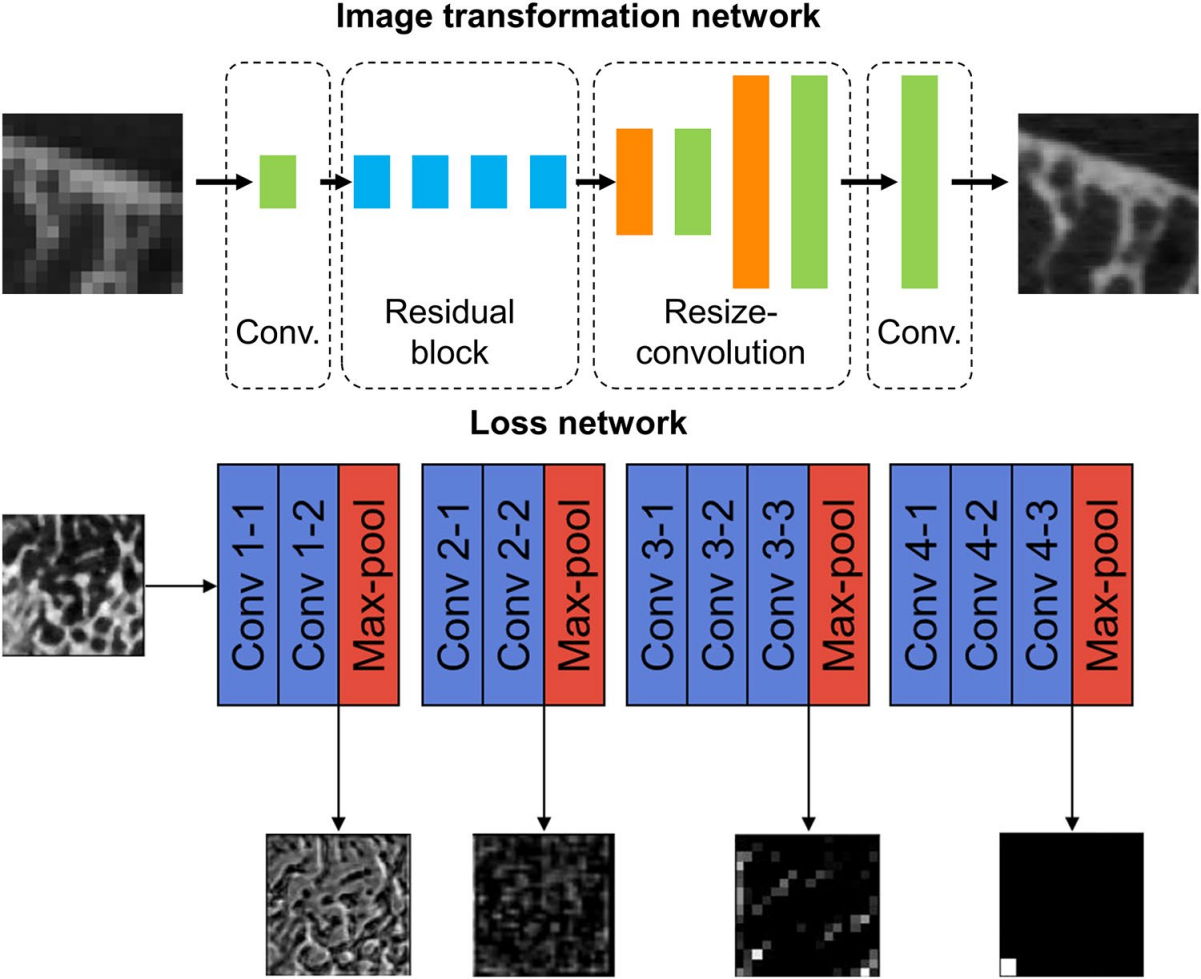
Top: The SR architecture used in the study. The architecture of Johnson et al. was modified by including resize-convolution layers instead of transposed convolutions. Bottom: The perceptual loss network was used in the visual model. Examples of perceptual loss network activations are shown for a trabecular bone reconstruction

The model architecture was inspired by Johnson et al. [46], including four residual blocks (Fig. 2, top). The transposed convolution layer was replaced by resize convolution [47]. The model was designed to yield a magnification factor of four. To conduct the training process, we used an in-house developed Collagen framework, a toolkit for reproducible machine-learning experiments (https://github.com/MIPT-Oulu/Collagen). We used three models, with a variety of five different loss functions in the experiments: (1) The baseline model utilized mean-squared error (MSE) and total variation (TV) as traditional pixel-wise losses, with respective weights of 0.8 and 0.2. (2) The structure model optimized the complement of the structure similarity index (SSIM), aiming to capture the bone microstructure. (3) The visual model combined mean absolute error (MAE), TV, and perceptual loss (PL), aiming to provide the best perceptual quality, using weights of 0.1, 1.0, and 1.0, respectively. Features from a pretrained VGG16 model were used as the PL (Fig. 2, bottom). The weights of the loss functions were chosen manually during the initial experiments of the study.

The models were trained using the Adam optimizer (parameters: *α* = 0.0001, *β* = 0.0001) for 50 epochs. The training was conducted under fourfold cross-validation, ensuring that the samples with the same patient ID were not mixed between the splits. During inference, the predictions were combined using a sliding window (16 × 16-pixel window with 8 × 8-pixel steps). A Gaussian kernel was applied to only focus the model predictions on the center of the tile, reducing the edge artifacts. To assess the performance of training, pixel-wise metrics (MSE, PSNR, SSIM) were calculated for the validation folds.

### Bone Microstructure Analysis

Morphological 3D parameters were quantified from the CBCT-imaged ex vivo test set, using conventional image processing, and SR. The true microstructure was analyzed using high-resolution µCT imaging. The volumes were binarized using the Otsu threshold [48]. An ad-hoc Python script was used to calculate the recommended morphological parameters: bone volume fraction (BV/TV), trabecular thickness (Tb.Th), trabecular separation (Tb.Sp), and trabecular number (Tb.N) [49]. In the case of the 2D models, the parameters were assessed for the axial 2D predictions as well as an average of the predictions of the three orthogonal planes. To provide benchmark comparisons, tricubic interpolation and an image processing-based pipeline were used. The image processing pipeline included multiple subsequent filters prior to the binary thresholding (anisotropic diffusion, contrast stretching, median filter). The results were compared using Pearson correlation. The 95% confidence intervals were estimated for the models that are trained on multiple random seeds. Finally, Bland-Altman analysis was conducted for the prediction of BV/TV using the reference methods and the best-performing super-resolution model.

### Clinical Validation Images

To assess the technical image quality, the spatial resolution was quantified from the reconstructed phantom images and SR predictions. This was achieved by estimating the MTF using the six-line pair patterns. The standard deviation was determined from a rectangular region of interest including each of the line pairs to provide a practical assessment of the function [13]. The frequency of 0.5 MTF (MTF_50%_) and 0.1 MTF (MTF_10%_), corresponding to a half-value and the limit of spatial resolution, are estimated from the graph.

To demonstrate the validity of the method in the clinical domain, we tested the models on multiple clinical imaging targets: ankle, knee, wrist, and dental CBCT. The predictions and interpolated CBCT images were compared visually. The reconstructions were normalized and converted from 16-bit to 8-bit images. To save memory and computational time, small volumes of interest were selected from the wrist and the ankle (wrist = 6.3 × 6 × 3.7 cm, ankle = 6.6 × 6.3 × 4.8 cm). For the knee scan, the full joint was processed (10 × 10 × 10 cm, output size = 1884 × 1932 × 1988 voxels) on the Puhti supercomputer (https://research.csc.fi/csc-s-servers). For the ankle, a lower resolution is used, and another set of models is trained (400 µm → 100 µm). In the case of knee, wrist and dental imaging, high-resolution models are used (200 µm → 50 µm).

The predictions and interpolations from the preoperative dental CBCT scans were assessed in a blinded reader study by an experienced dental radiologist (Reader 1) and dental surgeon (Reader 2) to grade the level of diagnostic quality. The Likert scale was used to score the signal-to-noise ratio, anatomical conspicuity (periodontal ligament space), image quality, artifacts, and diagnostic confidence of the images. The mean and standard deviation for the grades are reported and the inter-rater agreement is assessed using linearly weighed Cohen’s Kappa (*κ*). Finally, two µCT scans of the extracted teeth are coregistered with the clinical scans to allow a further visual comparison (Dataviewer, v. 1.5.6.2).

## Results

The conventional pixel-based performance metrics of training the 2D and 3D SR models on a 200 µm → 50 µm resolution scale are summarized in Table 2. The 2D baseline model (trained with MSE + TV loss) yields the highest performance (MSE = 0.0072 ± 0.0002, PSNR = 26.64 ± 0.07, SSIM = 0.812 ± 0.003). The 2D structure and visual models as well as the 3D baseline model yield slightly higher errors.

**Table 2:** Results on the out-of-fold validation for the 200 µm → 50 µm resolution models

Models	Out-of-fold evaluation		
	MSE	PSNR	SSIM
Baseline 2D	0.0072 ± 0.00003	**26.64 ± 0.014**	**0.812 ± 0.0005**
Baseline 3D	**0.0068 ± 0.0001**	24.8 ± 0.05	0.691 ± 0.002
Structure 2D	0.0084 ± 0.0001	25.5 ± 0.05	0.776 ± 0.006
Visual 2D	0.015 ± 0.007	25 ± 1.3	0.7 ± 0.06

The best performance on each metric is bolded

Experiments with different combinations of loss functions are listed with a two-dimensional (2D) or volumetric (3D) model. The value for the standard error of mean is reported after the mean value

*MSE* mean-squared error, *PSNR* peak signal-to-noise ratio, *SSIM* structure similarity index

### Ex Vivo Test Set: Prediction of Bone Microstructure

**Fig. 3:**
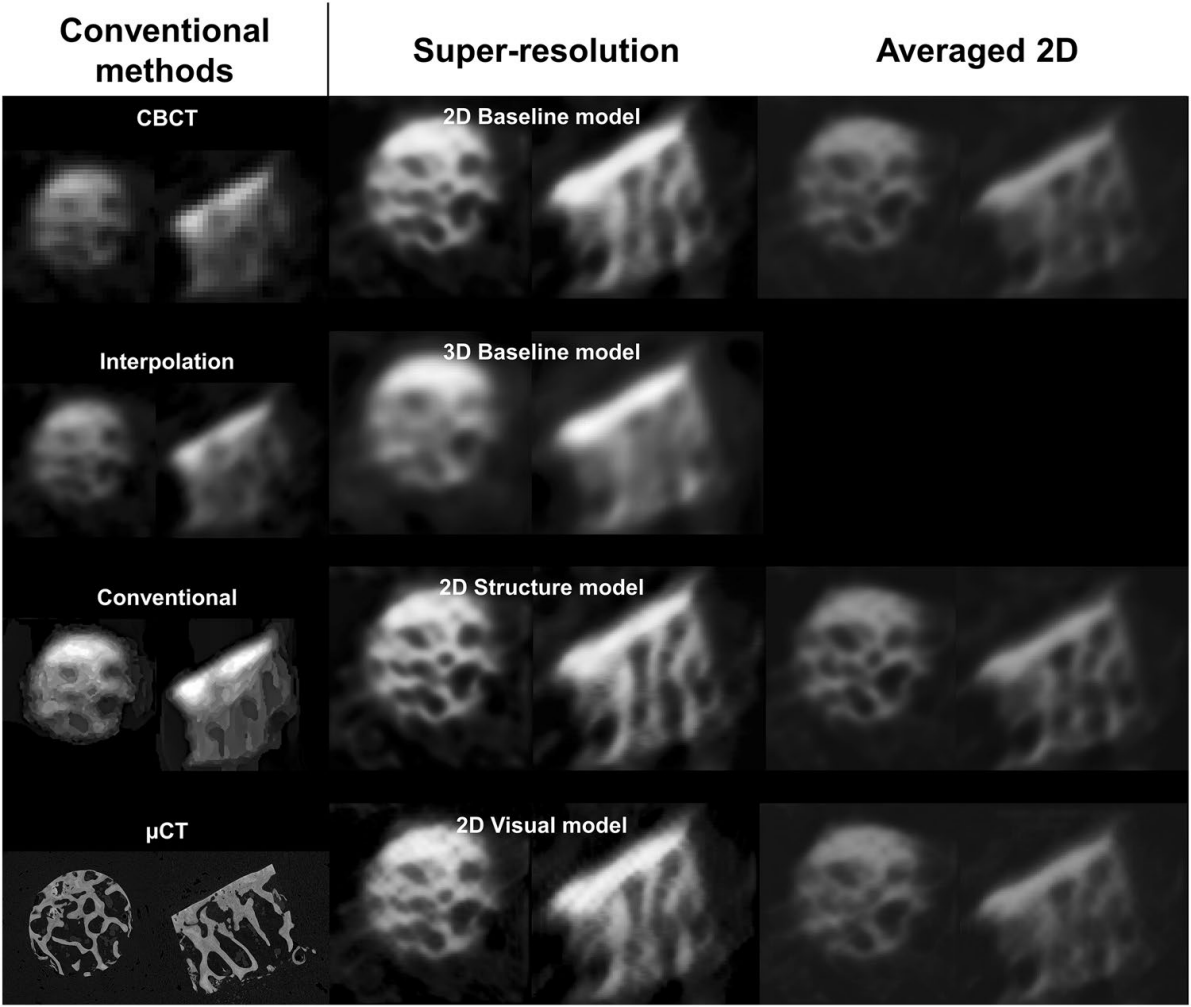
Comparison of conventional image quality improvement and super-resolution (SR) predictions on the osteochondral samples. The clearest structural definition is seen on the 2D models without averaging the three orthogonal planes.

The trained models were applied to the ex vivo test set to assess the performance of predicting the bone microstructure on unseen data (Table 3; Fig. 3; Figure, Online Resource 1 and 2). The 2D structure model yields the highest results (*r*_BVTV_ = 0.817 ± 0.005) and outperforms the interpolation (*r*_BVTV_ = 0.64) and conventional segmentation pipeline (*r*_BVTV_ = 0.67). A strong correlation is also observed with the 2D structure model for Tb.Sp (*r* = 0.756 ± 0.009). Bland-Altman analysis of BV/TV predictions resulted in a bias of 39.5% and 39.1% as well as standard deviation of 23.7% and 14.3% for conventional segmentation pipeline and structure model, respectively.

**Table 3:** Quantification of the bone parameters

Models	Averaging	Bone parameters			
		BV/TV	Tb.Th	Tb.Sp	Tb.N
Interpolation		0.64	0.34	0.59	− 0.4
Conventional segmentation		0.67	0.42	0.50	− 0.63
Baseline 2D	No	0.736 ± 0.006	0.404 ± 0.008	0.694 ± 0.004	− 0.514 ± 0.007
	Yes	0.665 ± 0.003	0.336 ± 0.003	0.608 ± 0.006	− 0.458 ± 0.0001
Structure 2D	No	**0.817 ± 0.005**	**0.53 ± 0.02**	**0.756 ± 0.009**	**− 0.489 ± 0.007**
	Yes	0.731 ± 0.007	0.436 ± 0.006	0.613 ± 0.010	− 0.41 ± 0.02
Visual 2D	No	0.758 ± 0.012	0.453 ± 0.011	0.70 ± 0.02	− 0.57 ± 0.02
	Yes	0.674 ± 0.004	0.340 ± 0.009	0.609 ± 0.011	− 0.5 ± 0.02
Baseline 3D		0.654 ± 0.010	0.33 ± 0.03	0.63 ± 0.011	− 0.34 ± 0.03

The highest correlation on each parameter is bolded﻿

Predictions from each model were binarized and the bone parameters were compared to the micro-computed tomography (µCT) ground truth. The values indicate Pearson correlations and the respective 95% confidence intervals

BV/TV = bone volume fraction, Tb.Th = trabecular thickness, Tb.Sp = trabecular separation, Tb.N = trabecular number

### Technical Image Quality

**Fig. 4:**
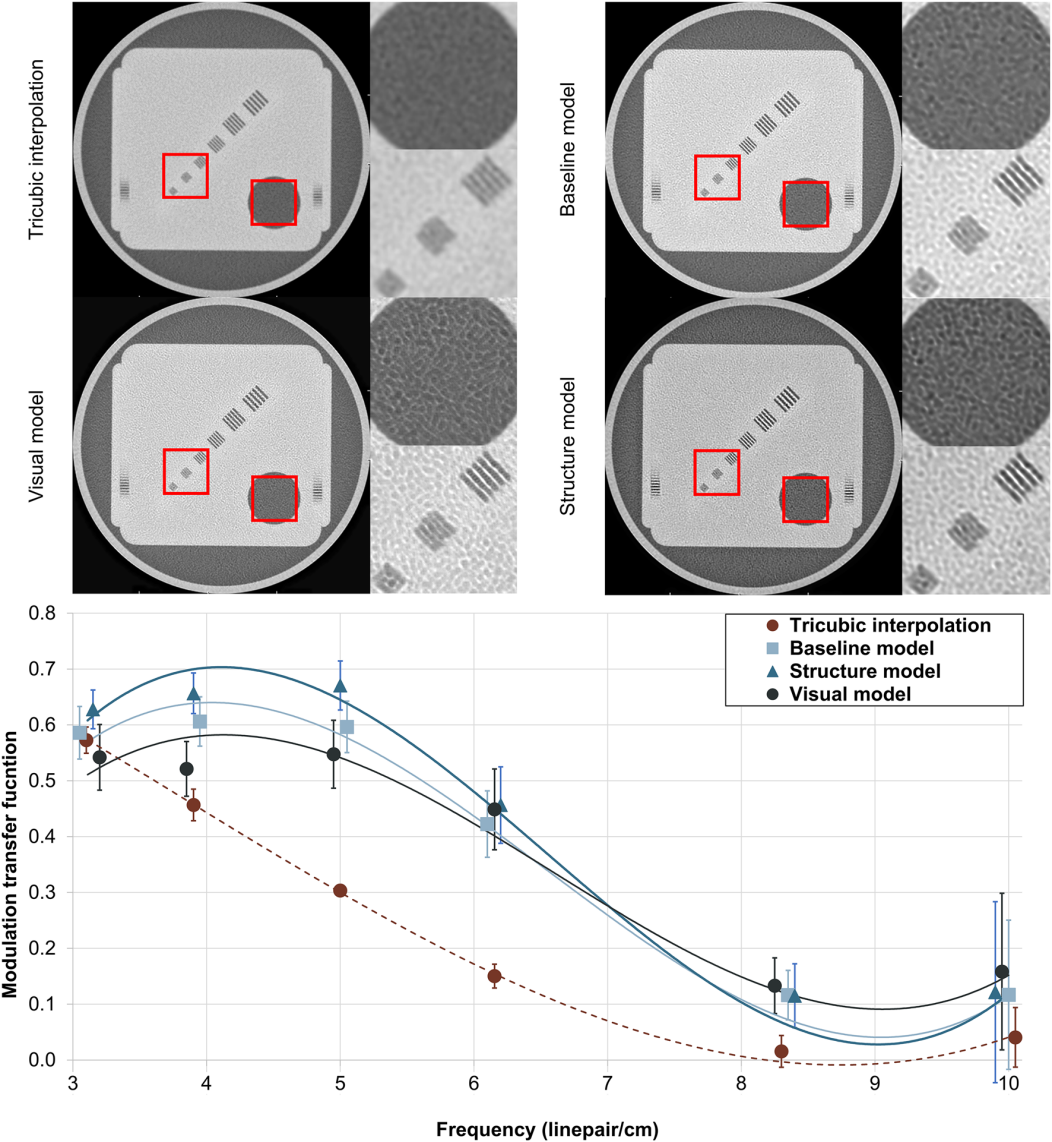
A clinical CT scan of a commercially available quality assurance phantom, with the corresponding interpolations and super-resolution (SR) predictions (top). Using the SR models, another set of line pairs can be distinguished from the CT slices. However, the perpendicular plane resolution is less improved. This can be seen as the number of diagonal lines on the edge of the phantom (that are averaged from multiple different depths) is not decreasing. The modulation-transfer functions (MTF) show that all the SR models provide an increase in spatial resolution (bottom). The 95% confidence intervals are shown for each MTF measurement. Rough trendlines of the MTFs are shown with a third-order polynomial fit

The technical image quality was determined by comparing interpolated and predicted clinical CT images from a quality assurance phantom. The fifth line pair pattern at 8.3 line pair per cm frequency can be visually resolved from the SR predictions but not from the interpolated image (Fig. 4a). Furthermore, the MTFs suggest a higher image quality in the predictions at the 4–8 line pairs per cm frequency range. An increase of 0.2 is seen between 5–6 line pairs per cm (Fig. 4b). Based on the estimated MTF curves, the interpolated CT images reach MTF_50%_ and MTF_10%_ at roughly 3.5 and 7.0 line pairs per cm, respectively. The MTF curves from the SR models reach the MTF_50%_ and MTF_10%_ values later, at 5.0 and 8.0 line pairs per cm. Standardization based on plexiglass and water grayscale values was not feasible for the SR models (Figure, Online Resource 3).

### Clinical Image Quality on Musculoskeletal Application

**Fig. 5:**
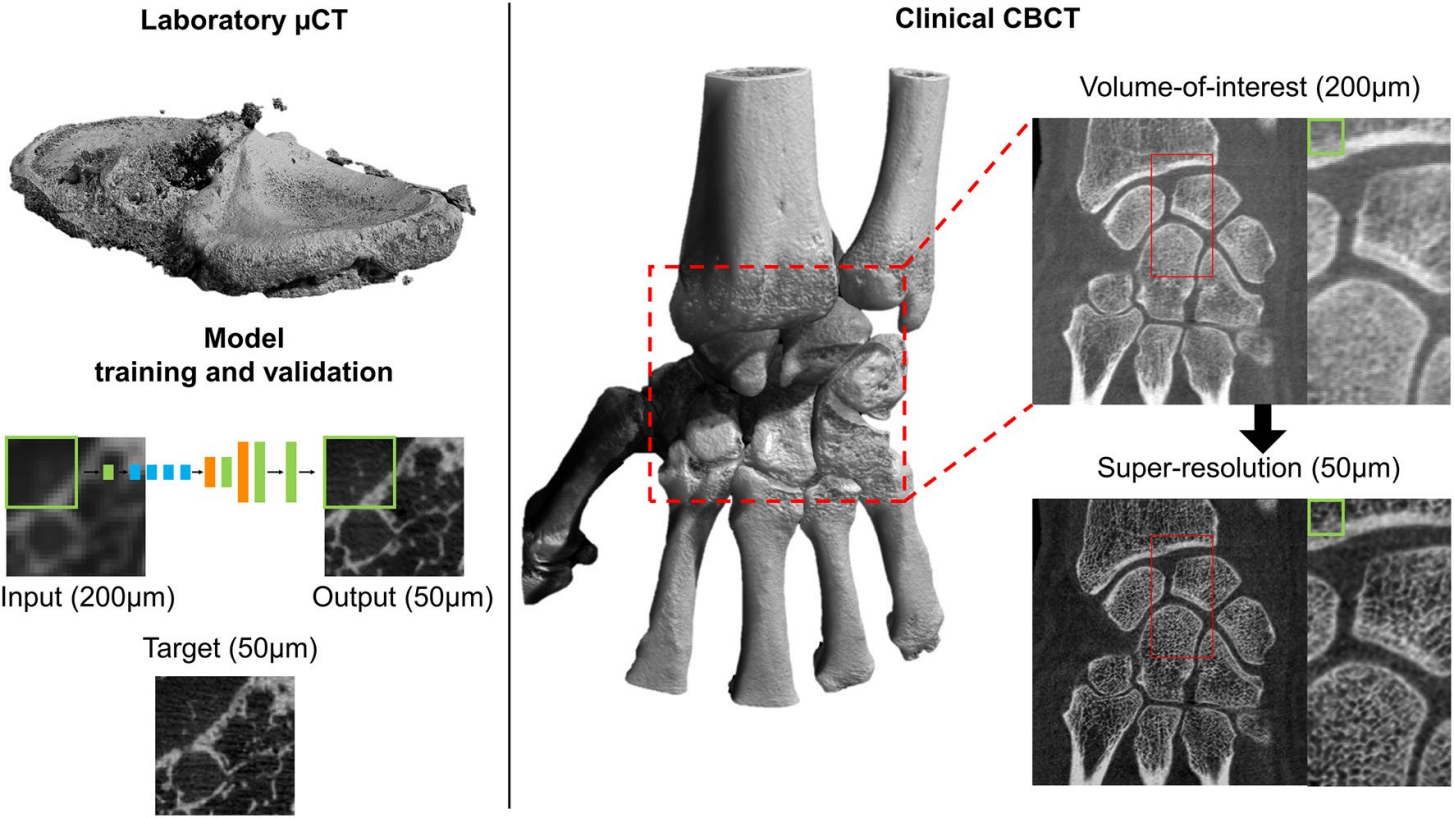
Overview of the proposed super-resolution (SR) method. Tissue blocks are scanned with micro-computed tomography (µCT) and used to train the model (left). The trained model can be utilized for clinical cone-beam CT (CBCT) images using a patch-by-patch sliding window, the size of one patch is depicted with a green rectangle. In this case, predictions from all orthogonal planes were averaged

**Fig. 6:**
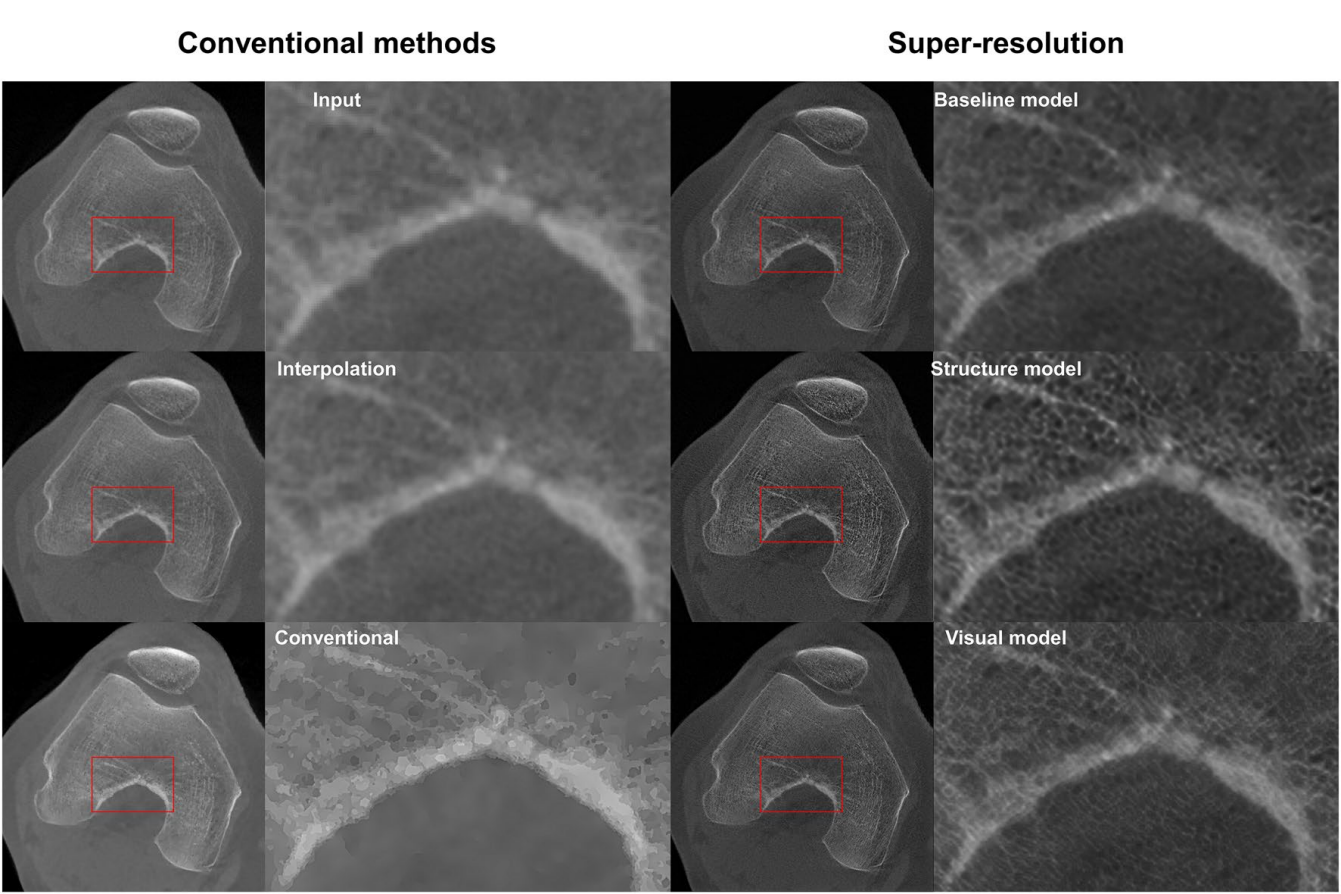
Comparison of conventional image quality improvement and super-resolution (SR) predictions on clinical scans of the knee joint. Predictions were conducted for the full joint; magnifications are shown to allow for a better visual comparison

An overview of the proposed SR method and an example of wrist SR are presented in Fig. 5. A volume of interest in the wrist joint was passed through the model to reduce the computational time. The inference computation on all three planes took roughly one hour on two graphical processing units (Nvidia GeForce GTX 1080 Ti). More structural details are visible in the prediction, but the cortical bone is visually too porous when compared to the original CBCT image. We also tested whether the inclusion of teeth images in training data changed the appearance, but only small differences were observed (Figure, Online Resource 4) compared to the original training setup. In the case of knee CBCT, a large volume was processed on the Puhti supercomputer. The 2D models were compared to the interpolation and conventional image processing pipeline (Fig. 6). The structural details were visually highlighted the best in the results from the baseline and structure models. The visual model created a flickering artifact in noisy and unclear regions of the tissue (Video, Online Resource 5).

**Fig. 7:**
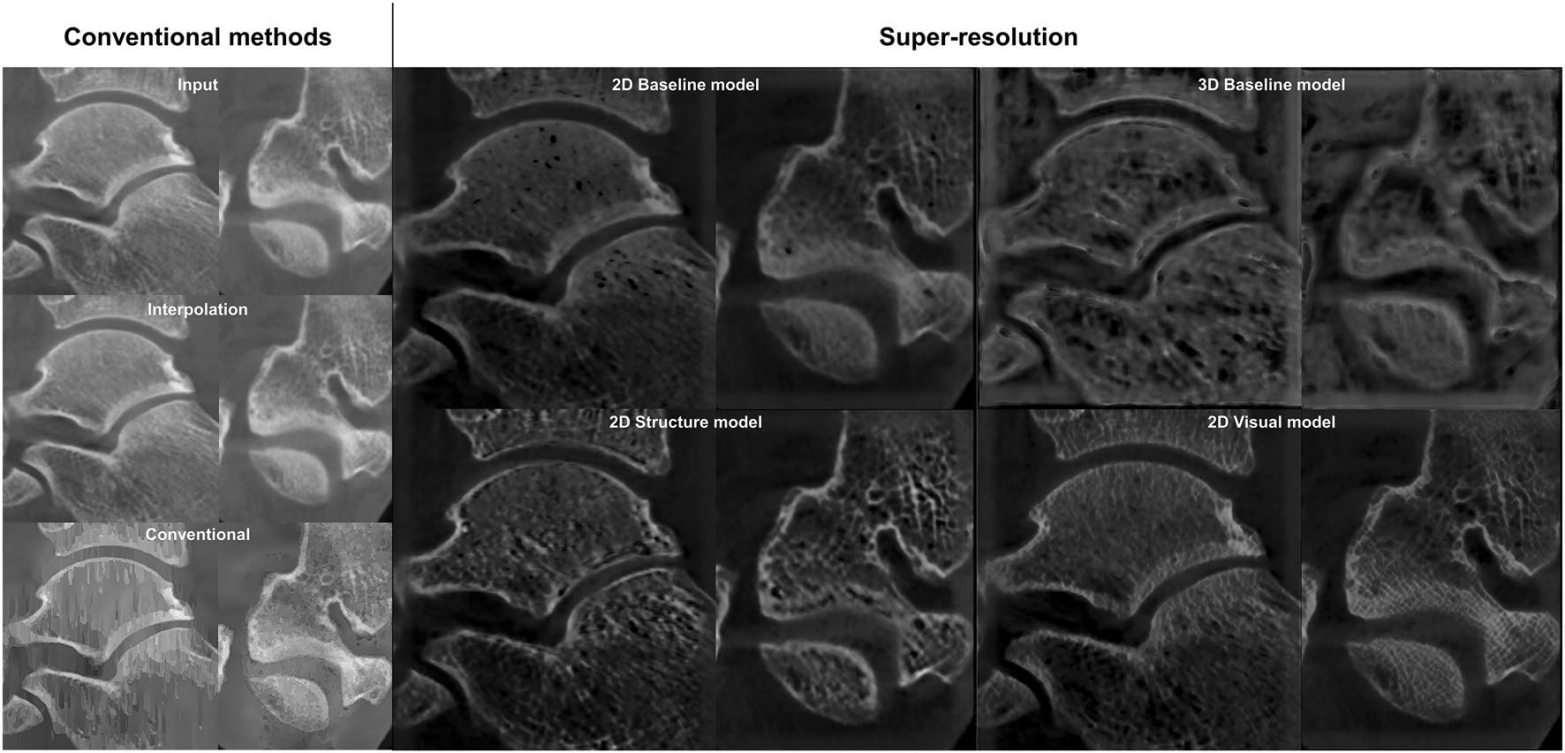
Comparison of conventional image quality improvement and super-resolution (SR) predictions on clinical scans of the ankle joint. The baseline 3D model provided the noisiest results

The ankle CBCT images were visually compared to interpolation, conventional image processing pipeline, as well as 2D and 3D predictions (Fig. 7). The 2D models show reduced noise and slightly more detail compared to the conventional methods. The most clearly visible structures were yielded by the structure model. The 3D baseline model converged to a solution with a slightly lower image quality. This led to more noisy prediction images, highlighting only the large-scale details.

### Clinical Image Quality on Dental Application

**Fig. 8:**
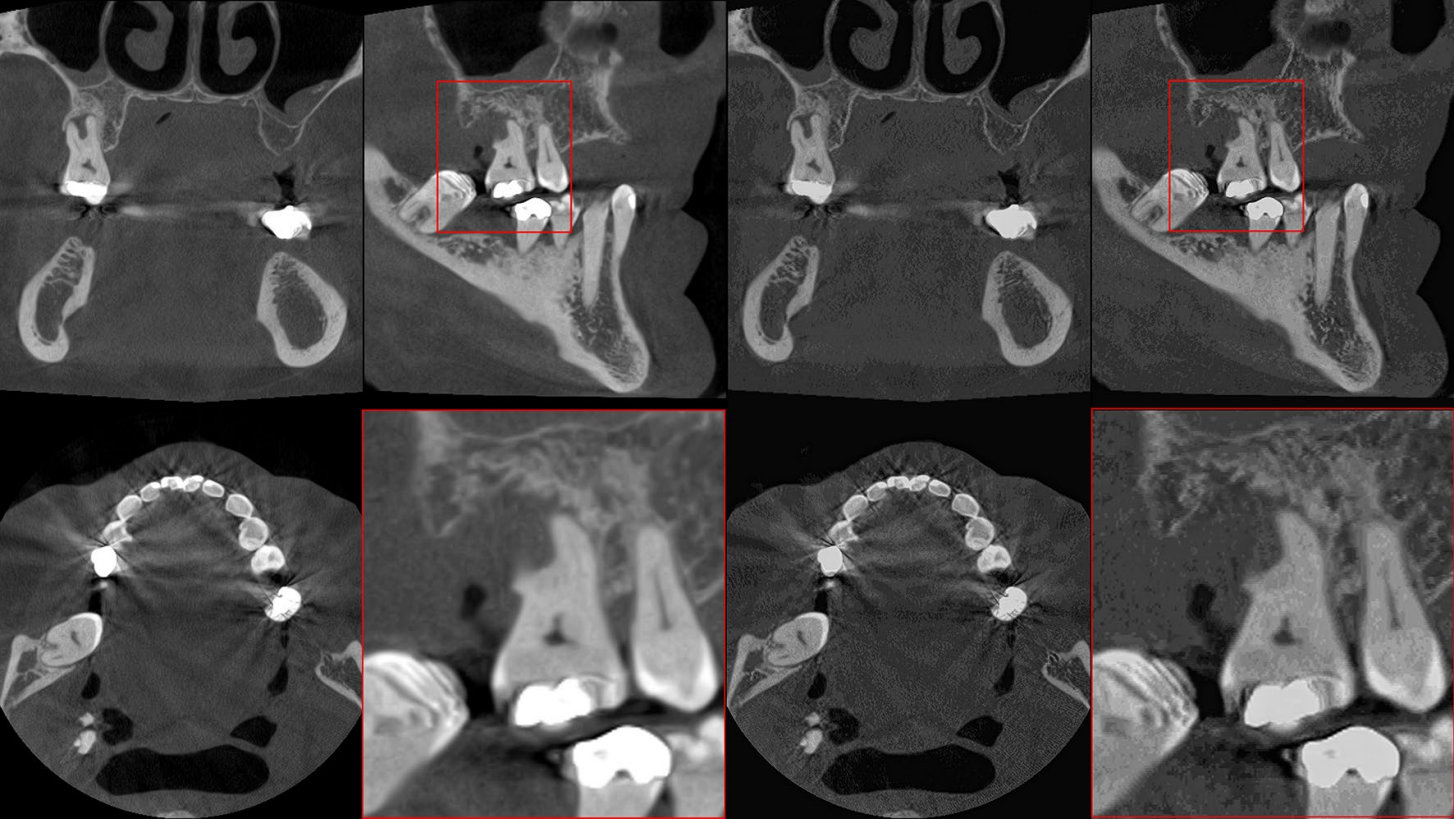
Examples of maxillofacial cone-beam CT images (left) and corresponding super-resolution predictions (right). Predictions are shown from the structure model, without averaging the orthogonal planes

An example of SR prediction on maxillofacial CBCT is shown in Fig. 8. In this case, the teeth of the patient were not used in training the SR model. A comparison of CBCT, SR, and µCT of extracted teeth from two other patients is illustrated in a Video, Online Resource 6. Small structures are better highlighted on the SR images compared to the original CBCT, and a previously unseen gap can be seen in the lamina dura next to the tooth that was removed from patient one (indicated with a red arrow). We noted artifacts from the SR algorithms especially within the enamel. The results of the reader study are described in Table 4. When accounting for Bonferroni correction, no significant differences were observed for scores of Reader 1, although a slight trend of higher scores towards the interpolated images was observed. Reader 2 scored higher signal-to-noise ratio, anatomical conspicuity, image quality, and diagnostic confidence for the baseline model compared to interpolation. The inter-rater agreement was slight (0.0–0.2) or fair (0.2–0.4), yet a substantial agreement was found for signal-to-noise ratio (0.64, visual model) and artifacts (0.80, baseline model).

**Table 4:** Blinded reader qualitative assessments

Model	Score (Mean + SD)					
	Signal-to-noise ratio	Anatomical conspicuity	Image quality	Artifacts	Diagnostic confidence	Overall average
Reader 1						
Interpolation	2.4 ± 0.7	2.9 ± 0.6	2.8 ± 0.4	2.7 ± 0.5	2.8 ± 0.4	2.7 ± 0.5
Baseline model	2.2 ± 0.4	2.7 ± 0.5	2.6 ± 0.5	2.7 ± 0.5	2.7 ± 0.5	2.6 ± 0.5
Structure model	1.9 ± 0.8	2.4 ± 0.5	2.4 ± 0.5	2.2 ± 0.4	2.4 ± 0.5	2.3 ± 0.6
Visual model	2.1 ± 0.3	2.4 ± 0.5	2.3 ± 0.5	2.3 ± 0.5	2.7 ± 0.5	2.4 ± 0.5
Reader 2						
Interpolation	1.4 ± 0.7	2.1 ± 0.8	1.8 ± 0.7	2.2 ± 1.1	1.8 ± 1.0	1.9 ± 0.9
Baseline model	2.4 ± 0.5*	2.8 ± 0.7*	2.8 ± 0.7*	2.8 ± 0.7	2.9 ± 0.6*	2.7 ± 0.6
Structure model	1.4 ± 0.5	2.0 ± 0.5	1.8 ± 0.7	2.1 ± 1.1	1.9 ± 0.8	1.8 ± 0.7
Visual model	2.0 ± 0.5	2.2 ± 0.4	2.0 ± 0.5	2.1 ± 0.6	2.1 ± 0.6	2.1 ± 0.5
Agreement (κ)						
Interpolation	0.147	0.241	0.047	0.077	0.039	
Baseline model	0.526	0.400	0.250	0.800	0.143	
Structure model	0.400	0.217	0.156	0.087	0.031	
Visual model	0.640	0.053	0.308	0.143	0.211	
95% CI						
Interpolation	(0.108–0.186)	(0.224–0.258)	(0.018–0.076)	(0.048–0.106)	(0.006–0.071)	
Baseline model	(0.518–0.534)	(0.389–0.411)	(0.238–0.262)	(0.793–0.807)	(0.132–0.153)	
Structure model	(0.379–0.421)	(0.202–0.233)	(0.135–0.178)	(0.060–0.114)	(0.010–0.051)	
Visual model	(0.636–0.644)	(0.043–0.062)	(0.298–0.317)	(0.132–0.153)	(0.196–0.225)	

The mean and standard deviation of the scores are given for each category. The inter-reader agreement was assessed using Cohen’s Kappa (κ) with 95% confidence intervals. Statistical significance for differences between interpolation and super-resolution (SR) was assessed using the Wilcoxon Signed Rank test (Bonferroni corrected for three comparisons) and is indicated with an asterisk (*)

*CI* confidence interval, *p < 0.05

## Discussion

In this study, we presented a deep learning-based super-resolution method to increase medical CBCT image quality in musculoskeletal and dental imaging domains and demonstrated how to validate the methods in several clinical domains. This study has several important contributions. First, the SR predictions were assessed for conventional image metrics on cross-validation, 3D bone microstructure quantification on the ex vivo test set, and the technical increase in spatial resolution using a quality assurance phantom. Second, the versatility of the SR algorithm was tested on clinical CBCT images of the wrist, knee, ankle, and maxillofacial region, and the dental image quality is quantified in a reader study, completely independent of the training process. This simulates deploying a method developed solely on limited laboratory data in the highly variable clinical environment, which we consider one of the key strengths of this study. Third, to facilitate further development of the musculoskeletal and dental imaging field, the source code of the project is published on GitHub (10.5281/zenodo.8041943) and the pretrained models used for dental SR predictions are available on Mendeley Data (10.17632/4xvx4p9tzv.1).

The out-of-fold validation results (Table 2) suggest that the 2D baseline model performs best and that the 3D baseline model yields the lowest performance. The analysis is based on traditional pixel-wise comparisons to high-resolution images. However, the analysis of osteochondral ex vivo samples shows that the 2D structure model is the best for predicting microstructural bone details (*r*_BVTV_ = 0.817 ± 0.005). Furthermore, averaging the prediction on three orthogonal planes did not improve the result. Likely, averaging the 2D predictions that do not account for adjacent slices causes smearing of the details, resulting in a lower correlation at least in the studied small four-millimeter samples. Finally, we would like to note that we also trained UNet and FPN segmentation models to predict the bone microstructure, but the models overfit and did not generalize from the training on the tissue blocks to the challenging ex vivo test set. Thus, we hypothesize that the SR method is more resistant to domain shift compared to DL segmentation. This is further supported by the multiple of applications presented using the same training data.

The results of the quality assurance phantom analysis suggested that the SR models increase CT spatial resolution, both visually and quantitatively. Importantly, we also noticed that the models heavily modified the grayscale distribution of the scan, and the values on the line pair pattern exceeded those in the uniform areas of the phantom. This eventually led us to scale the MTF curves, based on the maximum intensity of the scan (Figure, Online Resource 3). Importantly, the quantitative Hounsfield unit values are lost after processing, and the resulting prediction only describes the bone structure, not density or material composition. This is a potential limitation of patch-based super-resolution but could be alleviated in the future by using a wider dynamic range of training images or more complex SR models.

The experiments on the wrist, knee, ankle, and maxillofacial region reveal that the models generalize very well on different anatomical regions, although in some regions of cortical bone, there is a sudden increase in porosity, especially in the wrist images. This is likely a result of having a high amount of trabecular bone in the training data. However, this was not confirmed in the Figure, Online Resource 4, as there were no major differences in the images. In the maxillofacial region, our initial experiments included multiple artifacts near teeth, when using only the knee tissue blocks in training. Averaging the predictions in three orthogonal planes preserves the structure better in the perpendicular plane but might smear the details in case of morphological analysis. This is also supported by the Video, Online Resource 5, where a flickering artifact is seen on the sagittal plane.

The reader study resulted in quite modest scores for both interpolated images and SR predictions. A slight preference for interpolated images was observed for the scores of Reader 1, and Reader 2 scored the Baseline model slightly higher compared to other models or interpolation. The low overall scores are likely due to the fact that the high dynamic range of the original 12-bit CBCT images is lost. This could be potentially alleviated in the future by training the models on a higher dynamic range rather than the conventional eight bits which would also better allow studying HU values of model output. Also, the volume of extracted teeth is very small, resulting in a much smaller number of tooth images compared to the knee tissue blocks (Table 1), and thus, the current dataset is not optimal for training SR models for dental images.

While promising, maxillofacial images show that the small, mineralized structures are better seen on the SR predictions, and even previously unseen pathologies might be revealed (Video, Online Resource 6). However, we also noted definite artifacts within the enamel which could be confused for caries lesions. A more specialized training dataset would be crucial to alleviate such issues. Indeed, we expect that better results could be obtained in the future using a dataset with preclinical scans of entire cadaveric jawlines and soft tissues. Even more readily available animal models, such as pig maxillofacial tissue, could be considered to provide the SR model examples closer to the target distribution.

In medical diagnostics, it is imperative that the SR models do not induce biases from the training set and remove or add new diagnostic features to the predicted high-resolution images [45]. Upscaling the images poses, a serious theoretical problem: multiple visually distinct high-resolution images can downscale to the same low-resolution image [50]. This serious limitation warrants thorough validation experiments before SR can be utilized in the clinical environment. This would be an excellent area for future studies, where predictions of healthy tissue and small fractures or other pathological conditions could be analyzed in more detail. For example, the method could be retrospectively compared on datasets with follow-up information on specific pathological conditions, such as osteoporosis or osteoarthritis from musculoskeletal images or periodontal disease from maxillofacial images.

Despite being not specifically shown in the present study, we would hypothesize that models that generate entirely new images from a latent space, such as generative adversarial networks, could have a higher risk of “hallucinating” nonexistent pathological features, whereas a traditional CNN is more limited to modifying the original image, even though it is upscaled from low resolution. An interesting future improvement could be integrating an uncertainty map into the reconstruction, with a possible warning to the end-user, or merging the SR prediction with the original reconstruction in the uncertain areas of the image [51].

This study has several limitations. First, the best-performing 2D models did not account for changes in the perpendicular plane. An interesting future methodological improvement could include using a three-channel input image, including the adjacent slices. Second, most of the clinical comparisons presented in this study are restricted to qualitative or semiquantitative analysis. There are many studies where multiple radiologist readers assess the diagnostic image quality blindly from the SR and comparison images to show the increase in performance [37, 38, 52, 53]. Ideally, at least five readers should be included from different levels of education and experience [54, 55]. We would argue that the ratings provided by the radiologists are also somewhat subjective, and the true ground-truth information cannot be obtained in clinical studies without a subsequent tissue sample extraction. Third, the weights of the individual loss functions were chosen manually during the early experiments of this study. These should be ideally chosen using an ablation study or hyperparameter optimization. Finally, the SR prediction was conducted as post-processing rather than by directly reconstructing the projection images using deep learning. Indeed, the first CT vendors have already released reconstruction methods based on deep learning [29, 44]. As the projection data are often unavailable to the end-user, nonlinear CNN-based methods that work in the reconstruction domain could be more easily added, as an additional component to any CT scanner.

The deep-learning-enhanced medical images could have a high impact on the medical domain. The implications for the technology include higher patient throughput, more precise diagnostics, and disease interventions at an earlier state. The proposed SR can be directly applied to the existing clinical scans in the reconstruction domain and could, thus, have quality enhancement potential for routine hospital pipelines. Integration of SR methods in the hospital environment could facilitate a higher throughput, reducing the time radiologists need to reach a diagnosis on difficult cases as well as mitigating uncertainty in the diagnostic process. Radiologists could use the SR as an advanced “zoom” feature, analogous to how pathologists change the objective on a microscope. Training the models on laboratory data allows for pushing the spatial resolution limit further than what the clinical radiation doses or even the current CT technology would otherwise allow. Alternatively, the current image quality could be maintained with a lower dose which could increase the accessibility of CBCT and allow earlier diagnostic intervention.

## Citation Diversity Statement

Recent work in several fields of science has identified a bias in citation practices such that papers from women and other minority scholars are under-cited relative to the number of such papers in the field [56–60]. Here, we sought to proactively consider choosing references that reflect the diversity of the field in thought, form of contribution, gender, race, ethnicity, and other factors. First, we obtained the predicted gender of the first and last author of each reference by using databases that store the probability of a first name being carried by a woman [58, 61]. By this measure and excluding self-citations to the first and last authors of our current paper), our references contain 6.96% woman(first)/woman(last), 9.09% man/woman, 18.57% woman/man, and 65.38% man/man. This method is limited in that (a) names, pronouns, and social media profiles used to construct the databases may not, in every case, be indicative of gender identity and (b) it cannot account for intersex, non-binary, or transgender people. Second, we obtained predicted racial/ethnic category of the first and last authors of each reference by databases that store the probability of a first and last name being carried by an author of color [62, 63]. By this measure (and excluding self-citations), our references contain 16.38% author of color (first)/author of color(last), 12.83% white author/author of color, 22.97% author of color/white author, and 47.82% white author/white author. This method is limited in that (a) names and Florida Voter Data to make the predictions may not be indicative of racial/ethnic identity, and (b) it cannot account for Indigenous and mixed-race authors, or those who may face differential biases due to the ambiguous racialization or ethnicization of their names. We look forward to future work that could help us to better understand how to support equitable practices in science.

### Supplementary Information

Below is the link to the electronic supplementary material.

Online resource 1. Scatter plots of bone microstructural parameters obtained from the original high-resolution µCT images (x-axis) and the parameters measured from the low-resolution data (y-axis). Perfect agreement is indicated with a dashed line. All methods deviate from the perfect agreement, on the challenging test set, but the best results are obtained with the structure model for BV/TV and Tb.Sp. Supplementary file1 (TIF 6603 kb)

Online resource 2. Bland-Altman analysis for the measurement of BV/TV with interpolation, conventional segmentation pipeline and structure model. The continuous grey line indicates bias, and the dashed lines indicate 95% limits of agreement. Supplementary file2 (TIF 2323 kb)

Online Resource 3. The modulation transfer functions (MTF) are scaled based on the average of plexiglass and water in a region of interest. The super-resolution model’s predictions highlight the structures in the line pair patterns, and the grayscale values exceed the ones in smooth areas of plexiglass. This results in MTF values that exceed one. However, the results also show the effect of highlighting small structures better than the scaling used for Figure 4. Supplementary file3 (TIF 1819 kb)

Online Resource 4. Comparison of using knee tissue blocks and extracted teeth in training data. Structure model predictions are shown above. Only very small differences are seen between the images, suggesting that adding dental images did not improve the prediction accuracy of musculoskeletal cone-beam CT. Supplementary file4 (TIF 7379 kb)

Online Resource 5. Sagittal view of the knee. As the predictions are only created from the transaxial plane, a flickering artifact can be seen on the sagittal view. Supplementary file5 (MP4 168014 kb)

Online Resource 6. Maxillofacial cone-beam CT images of two patients, corresponding structure and baseline model predictions as well as micro-computed tomography (µCT) scans of the extracted teeth. Details are better preserved on the super-resolution prediction. A possible small gap is seen on the lamina dura of patient one, indicated with a red arrow. The tooth next to the tissue is later extracted and the corresponding µCT reconstruction is shown. Supplementary file6 (MP4 8635 kb)

## Data Availability

Data generated or analyzed during the study are available from the corresponding author by reasonable request.

## Code Availability

The source code and pretrained models for the study are publicly available.

## References

[1] Nieminen MT, Casula V, Nevalainen MT, Saarakkala S (2019). Osteoarthritis year in review 2018: imaging. Osteoarthr. Cartil..

[2] Roemer FW, Demehri S, Omoumi P, Link TM, Kijowski R, Saarakkala S (2020). State of the art: imaging of osteoarthritis—revisited 2020. Radiology.

[3] Law CP, Chandra RV, Hoang JK, Phal PM (2011). Imaging the oral cavity: key concepts for the radiologist. Br. J. Radiol..

[4] Roemer FW, Engelke K, Li L, Laredo JD, Guermazi A (2023). MRI underestimates presence and size of knee osteophytes using CT as a reference standard. Osteoarthr. Cartil..

[5] Ibad HA, de Cesar Netto C, Shakoor D, Sisniega A, Liu SZ, Siewerdsen JH (2023). Computed tomography: state-of-the-art advancements in musculoskeletal imaging. Invest. Radiol..

[6] Segal NA, Li S (2022). WBCT and its evolving role in OA research and clinical practice. Osteoarthr. Imaging.

[7] Schulze RKW, Drage NA (2020). Cone-beam computed tomography and its applications in dental and maxillofacial radiology. Clin. Radiol..

[8] Vitéz S, Kovács B, Ederer J, Schulte A-C, Partovi S, Bilecen D (2021). Cone beam CT for identifying fractures of the wrist and hand—an alternative to plain radiography?. Trauma.

[9] Veiga C, McClelland J, Moinuddin S, Lourenço A, Ricketts K, Annkah J (2014). Toward adaptive radiotherapy for head and neck patients: feasibility study on using CT-to-CBCT deformable registration for “dose of the day” calculations. Med. Phys..

[10] Zachiu C, de Senneville BD, Tijssen RHN, Kotte ANTJ, Houweling AC, Kerkmeijer LGW (2018). Non-rigid CT/CBCT to CBCT registration for online external beam radiotherapy guidance. Phys. Med. Biol..

[11] Posadzy M, Desimpel J, Vanhoenacker F (2018). Cone beam CT of the musculoskeletal system: clinical applications. Insights Imaging.

[12] Brüllmann D, Schulze RKW (2014). Spatial resolution in CBCT machines for dental/maxillofacial applications—what do we know today?. Dentomaxillofac. Radiol..

[13] Droege RT, Morin RL (1982). A practical method to measure the MTF of CT scanners. Med. Phys..

[14] Verdun FR, Racine D, Ott JG, Tapiovaara MJ, Toroi P, Bochud FO (2015). Image quality in CT: from physical measurements to model observers. Physica Medica.

[15] Huda W, Abrahams RB (2015). X-ray-based medical imaging and resolution. Am. J. Roentgenol..

[16] Anam C, Fujibuchi T, Budi WS, Haryanto F, Dougherty G (2018). An algorithm for automated modulation transfer function measurement using an edge of a PMMA phantom: impact of field of view on spatial resolution of CT images. J. Appl. Clin. Med. Phys..

[17] Friedman SN, Cunningham IA (2008). A moving slanted-edge method to measure the temporal modulation transfer function of fluoroscopic systems. Med. Phys..

[18] Ibrahim N, Parsa A, Hassan B, van der Stelt P, Wismeijer D (2013). Diagnostic imaging of trabecular bone microstructure for oral implants: a literature review. Dentomaxillofac. Radiol..

[19] Finnilä MAJ, Thevenot J, Aho O-M, Tiitu V, Rautiainen J, Kauppinen S (2017). Association between subchondral bone structure and osteoarthritis histopathological grade. J. Orthop. Res..

[20] Adams JE (2013). Advances in bone imaging for osteoporosis. Nat. Rev. Endocrinol..

[21] Genant HK, Engelke K, Prevrhal S (2008). Advanced CT bone imaging in osteoporosis. Rheumatology.

[22] Chu CR, Williams AA, Coyle CH, Bowers ME (2012). Early diagnosis to enable early treatment of pre-osteoarthritis. Arthr. Res. Ther..

[23] Karhula SS, Finnilä MAJ, Rytky SJO, Cooper DM, Thevenot J, Valkealahti M (2020). Quantifying subresolution 3D morphology of bone with clinical computed tomography. Ann. Biomed. Eng..

[24] He R-T, Tu M-G, Huang H-L, Tsai M-T, Wu J, Hsu J-T (2019). Improving the prediction of the trabecular bone microarchitectural parameters using dental cone-beam computed tomography. BMC Med. Imaging.

[25] Kemp P, Van Stralen J, De Graaf P, Berkhout E, Van Horssen P, Merkus P (2020). Cone-beam CT compared to multi-slice CT for the diagnostic analysis of conductive hearing loss: a feasibility study. J. Int. Adv. Otol..

[26] Beister M, Kolditz D, Kalender WA (2012). Iterative reconstruction methods in X-ray CT. Physica Medica.

[27] Geyer LL, Schoepf UJ, Meinel FG, Nance JW, Bastarrika G, Leipsic JA (2015). State of the art: iterative CT reconstruction techniques. Radiology.

[28] Thibault J-B, Sauer KD, Bouman CA, Hsieh J (2007). A three-dimensional statistical approach to improved image quality for multislice helical CT. Med. Phys..

[29] Greffier J, Frandon J, Si-Mohamed S, Dabli D, Hamard A, Belaouni A (2022). Comparison of two deep learning image reconstruction algorithms in chest CT images: a task-based image quality assessment on phantom data. Diagn. Interv. Imaging.

[30] Szczykutowicz TP, Toia GV, Dhanantwari A, Nett B (2022). A review of deep learning CT reconstruction: concepts, limitations, and promise in clinical practice. Curr. Radiol. Rep..

[31] Panda J, Meher S (2022). An improved Image Interpolation technique using OLA e-spline. Egyptian Inform. J..

[32] Fang L, Monroe F, Novak SW, Kirk L, Schiavon CR, Yu SB (2021). Deep learning-based point-scanning super-resolution imaging. Nat. Methods.

[33] You C, Li G, Zhang Y, Zhang X, Shan H, Li M (2020). CT super-resolution GAN constrained by the identical, residual, and cycle learning ensemble (GAN-CIRCLE). IEEE Trans. Med. Imaging.

[34] Isola, P., J.-Y. Zhu, T. Zhou, and A. A. Efros. Image-to-image translation with conditional adversarial networks. In: Proceedings—30th IEEE Conference on Computer Vision and Pattern Recognition, CVPR 2017, 2017, pp. 5967–5976. 10.1109/CVPR.2017.632

[35] Zhu, J.-Y., T. Park, P. Isola, and A. A. Efros. Unpaired image-to-image translation using cycle-consistent adversarial networks. In: Proceedings of the IEEE International Conference on Computer Vision, 2017. pp. 2242–2251. 10.1109/ICCV.2017.244

[36] Chaudhari AS, Fang Z, Kogan F, Wood J, Stevens KJ, Gibbons EK (2018). Super-resolution musculoskeletal MRI using deep learning. Magn. Reson. Med..

[37] Chaudhari AS, Stevens KJ, Wood JP, Chakraborty AK, Gibbons EK, Fang Z (2020). Utility of deep learning super-resolution in the context of osteoarthritis MRI biomarkers. J. Magn. Reson. Imaging.

[38] Rudie JD, Gleason T, Barkovich MJ, Wilson DM, Shankaranarayanan A, Zhang T (2022). Clinical assessment of deep learning–based super-resolution for 3D volumetric brain MRI. Radiol. Artif. Intell..

[39] Li, H., R. G. N. Prasad, A. Sekuboyina, C. Niu, S. Bai, W. Hemmert, et al. Micro-Ct synthesis and inner ear super resolution via generative adversarial networks and bayesian inference. In: 2021 IEEE 18th international symposium on biomedical imaging (ISBI), 2021, pp. 1500–1504. 10.1109/ISBI48211.2021.9434061

[40] Yu H, Wang S, Fan Y, Wang G, Li J, Liu C (2022). Large-factor Micro-CT super-resolution of bone microstructure. Front. Phys..

[41] Zhang, Q., Y. N. Wu, and S.-C. Zhu. Interpretable convolutional neural networks. In: 2018 IEEE/CVF Conference on Computer Vision and Pattern Recognition, 2018, pp. 8827–8836. 10.1109/CVPR.2018.00920

[42] Selvaraju, R. R., M. Cogswell, A. Das, R. Vedantam, D. Parikh, and D. Batra. Grad-CAM: visual explanations from deep networks via gradient-based localization. In: Proceedings of the IEEE International Conference on Computer Vision, 2017.pp. 618–626. 10.1109/ICCV.2017.74

[43] Ribeiro, M. T., S. Singh, and C. Guestrin. ‘Why should i trust you?’ Explaining the predictions of any classifier. In: Proceedings of the ACM SIGKDD International Conference on Knowledge Discovery and Data Mining, 2016, pp. 1135–1144. 10.1145/2939672.2939778

[44] Tsujioka K, Yamada K, Niwa M (2022). Performance evaluation of micro-vessels imaging by deep learning reconstruction targeting ultra-high-resolution CT (UHR-CT). J. Med. Imaging Radiat. Sci..

[45] Colbrook MJ, Antun V, Hansen AC (2022). The difficulty of computing stable and accurate neural networks: on the barriers of deep learning and Smale’s 18th problem. Proc. Natl. Acad. Sci..

[46] Johnson, J., A. Alahi, and L. Fei-Fei. Perceptual losses for real-time style transfer and super-resolution. In: European Conference on Computer Vision, 2016. 10.1007/978-3-319-46475-6_43

[47] Odena, A., Dumoulin, V., and C. Olah. Deconvolution and checkerboard artifacts. *Distill* 2016http://distill.pub/2016/deconv-checkerboard/

[48] Otsu N (1979). A threshold selection method from gray-level histograms. IEEE Trans. Syst. Man Cybern..

[49] Bouxsein ML, Boyd SK, Christiansen BA, Guldberg RE, Jepsen KJ, Müller R (2010). Guidelines for assessment of bone microstructure in rodents using micro–computed tomography. J. Bone Miner. Res..

[50] Menon, S., A. Damian, S. Hu, N. Ravi, and C. Rudin. PULSE: self-supervised photo upsampling via latent space exploration of generative models. In: Proceedings of the IEEE Computer Society Conference on Computer Vision and Pattern Recognition, 2020, pp. 2434–2442. 10.1109/CVPR42600.2020.00251

[51] Zhang X, Sisniega A, Zbijewski WB, Lee J, Jones CK, Wu P (2023). Combining physics-based models with deep learning image synthesis and uncertainty in intraoperative cone-beam CT of the brain. Med. Phys..

[52] Chaika M, Afat S, Wessling D, Afat C, Nickel D, Kannengiesser S (2023). Deep learning-based super-resolution gradient echo imaging of the pancreas: improvement of image quality and reduction of acquisition time. Diagn. Interv. Imaging.

[53] Van Dyck P, Smekens C, Vanhevel F, De Smet E, Roelant E, Sijbers J (2020). Super-resolution magnetic resonance imaging of the knee using 2-dimensional turbo spin echo imaging. Invest. Radiol..

[54] Obuchowski NA, Bullen J (2022). Multireader diagnostic accuracy imaging studies: fundamentals of design and analysis. Radiology.

[55] Gennaro G (2018). The, “perfect” reader study. Eur. J. Radiol..

[56] Caplar N, Tacchella S, Birrer S (2017). Quantitative evaluation of gender bias in astronomical publications from citation counts. Nat. Astron..

[57] Dion ML, Sumner JL, Mitchell SM (2018). Gendered citation patterns across political science and social science methodology fields. Polit. Anal..

[58] Dworkin JD, Linn KA, Teich EG, Zurn P, Shinohara RT, Bassett DS (2020). The extent and drivers of gender imbalance in neuroscience reference lists. bioRxiv.

[59] Mitchell SM, Lange S, Brus H (2013). Gendered citation patterns in international relations journals. Int. Stud. Perspect..

[60] Maliniak D, Powers R, Walter BF (2013). The gender citation gap in international relations. Int. Organ.

[61] Zhou D, Cornblath EJ, Stiso J, Teich EG, Dworkin JD, Blevins AS (2020). Sci.

[62] Ambekar, A., C. Ward, J. Mohammed, S. Male, and S. Skiena. Name-ethnicity classification from open sources. In: Proceedings of the 15th ACM SIGKDD international conference on Knowledge Discovery and Data Mining, 2009, pp. 49–58.

[63] Sood, G., and S. Laohaprapanon. Predicting race and ethnicity from the sequence of characters in a name 2018. arXiv:1805.02109

