# Supplementary figures and images for: Clinical Super-Resolution Computed Tomography of Bone Microstructure: Application in Musculoskeletal and Dental Imaging
Source: Ann Biomed Eng. 2024 Feb 15;52(5):1255–69. doi: 10.1007/s10439-024-03450-y (PMC10995025; doi:10.1007/s10439-024-03450-y)

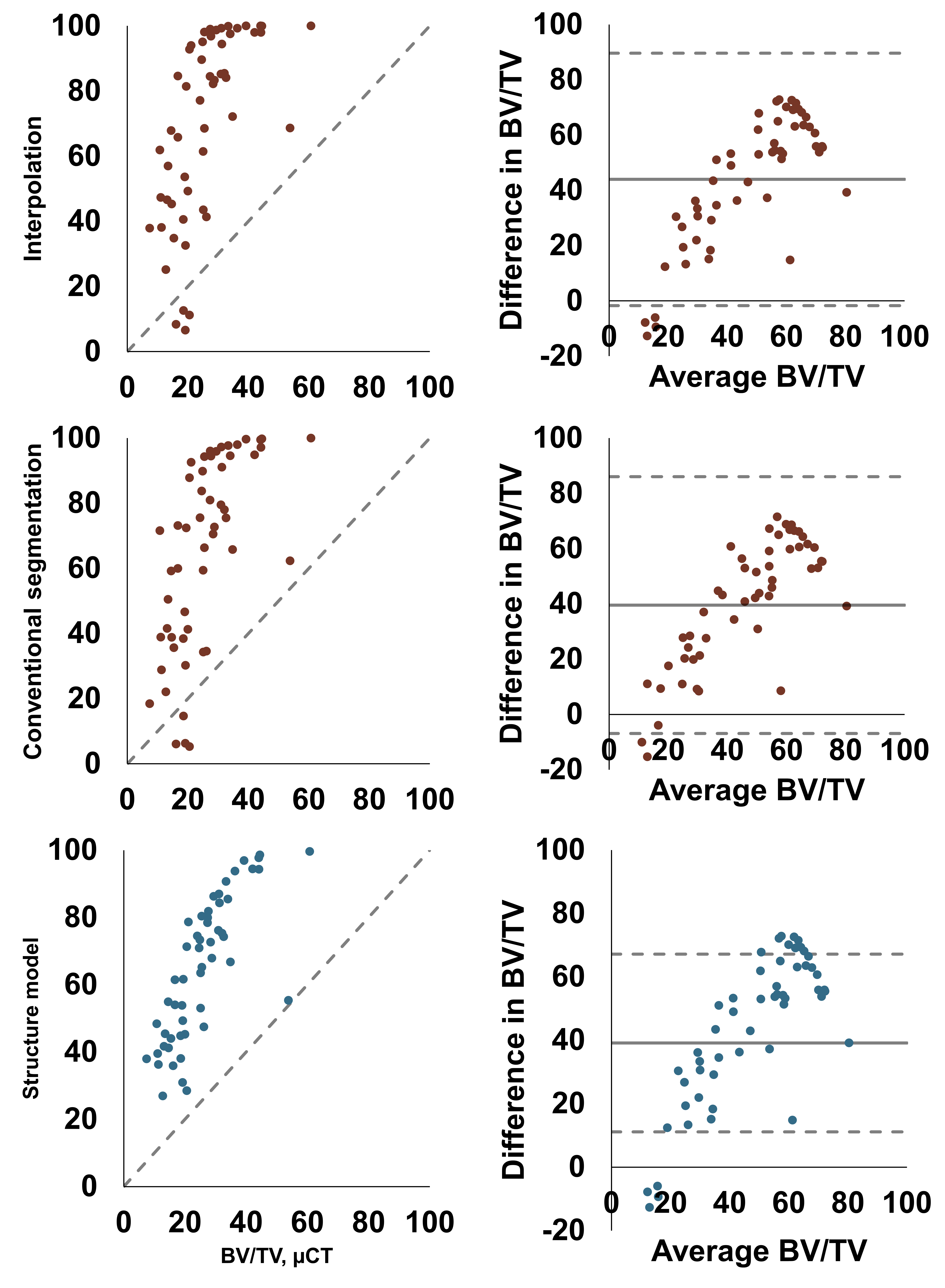

Supplement: Supplementary file 2 — Online resource 2. Bland-Altman analysis for the measurement of BV/TV with interpolation, conventional segmentation pipeline and structure model. The continuous grey line indicates bias, and the dashed lines indicate 95% limits of agreement. Supplementary file2 (TIF 2323 kb) [file 10439_2024_3450_MOESM2_ESM.tif]

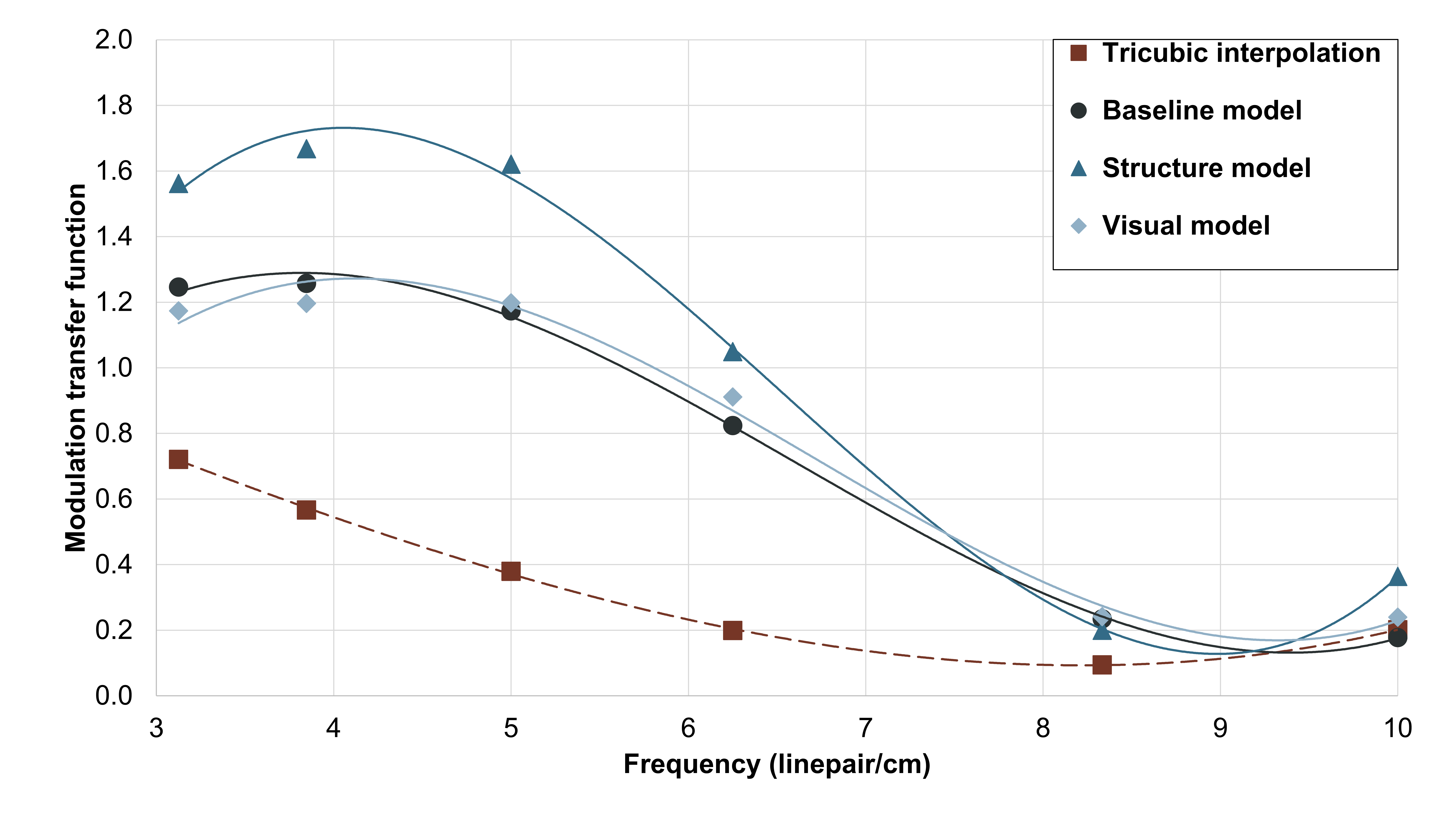

Supplement: Supplementary file 3 — Online Resource 3. The modulation transfer functions (MTF) are scaled based on the average of plexiglass and water in a region of interest. The super-resolution model’s predictions highlight the structures in the line pair patterns, and the grayscale values exceed the ones in smooth areas of plexiglass. This results in MTF values that exceed one. However, the results also show the effect of highlighting small structures better than the scaling used for Figure 4. Supplementary file3 (TIF 1819 kb) [file 10439_2024_3450_MOESM3_ESM.tif]

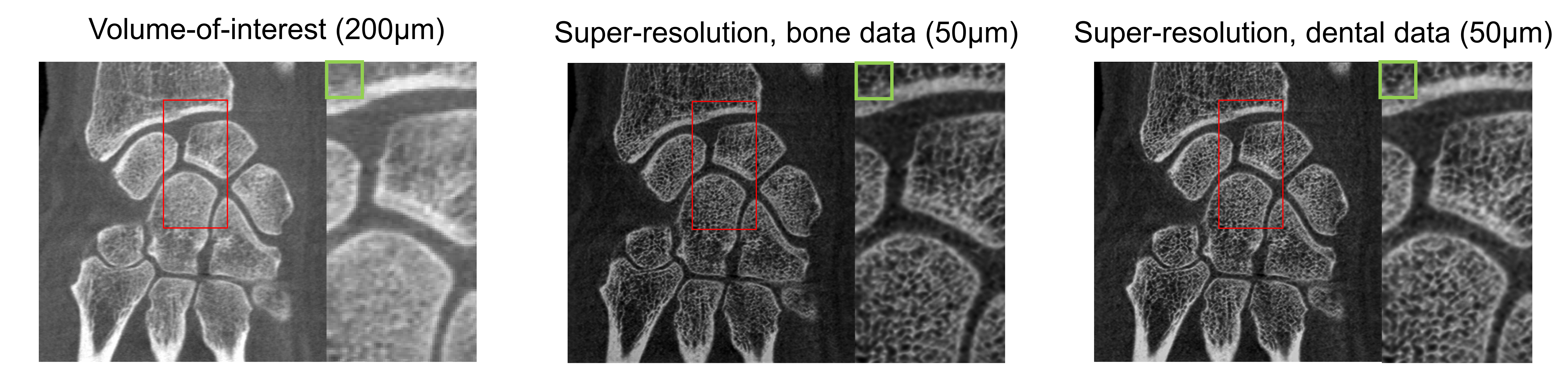

Supplement: Supplementary file 4 — Online Resource 4. Comparison of using knee tissue blocks and extracted teeth in training data. Structure model predictions are shown above. Only very small differences are seen between the images, suggesting that adding dental images did not improve the prediction accuracy of musculoskeletal cone-beam CT. Supplementary file4 (TIF 7379 kb) [file 10439_2024_3450_MOESM4_ESM.tif]
